# Can neurotechnology revolutionize cognitive enhancement?

**DOI:** 10.1371/journal.pbio.3002831

**Published:** 2024-10-29

**Authors:** Ines R. Violante, Prince Okyere

**Affiliations:** 1 School of Psychology, Faculty of Health and Medical Sciences, University of Surrey, Guildford, United Kingdom; 2 School of Biomedical Engineering and Imaging Sciences, King’s College London, London, United Kingdom

## Abstract

The development and implementation of neurotechnology for cognitive enhancement could spearhead a new wave of innovation. The authors argue in this Perspective that this will only happen with a more fundamental understanding of human brain function.

## Teaser

The development and implementation of neurotechnology for cognitive enhancement could spearhead a new wave of innovation in the information age. However, we argue here that this will only happen with a more fundamental understanding of human brain function.

*“Every technology is actually an artificial extension of the innate tendency possessed by all living beings to gain mastery over their environment, or at least not to surrender to it in their struggle for survival.”* Stanisław Lem, Summa Technologiae [1].

One of the biggest allures of neurotechnology is the possibility to enhance cognitive abilities. Cognition can be defined as the process by which organisms acquire, store, organize, and manipulate information and use it to guide behavior and decision-making. Thus, any singular or collective enhancement of cognitive function could provide a competitive advantage, which is a source of both promise and concern [2].

Cognitive enhancement has a long history. Its pursuit was first materialized through the accumulation and transfer of knowledge. The Pythagorean school shaped mathematics into a fundamental science and Mendel’s scientific publications planted the seed for the gene editing therapies now being applied to cure diseases. Other traditional forms of cognitive enhancement include caffeine and prescription stimulants, physical exercise, improvements in sleep quality, and memory training. Can these be boosted or replaced by technology? Efforts so far have not yet resulted in significant gains, with most technological attempts at cognitive enhancement producing small effect sizes obtained typically under laboratory conditions [3,4]. However, timid beginnings apply to technologies at the dawn of new eras. After all, the first mechanical vehicles were slower than horse-driven ones and the first consumer mobile phones were attached to cars. What is then missing for a leap in neurotechnology to achieve cognitive enhancement? In our opinion, we are reaching the tip of what can be developed through trial and error. Our view is that a leap in neurotechnology for neuroenhancement can only be achieved if it progresses alongside a more fundamental understanding of human brain function. As Lem put it “… *technological empiricism develops as far as it can*. *Edison tried to invent something like an “atomic engine”*, *but this did not—and could not—come to much because*, *whereas a dynamo can be built through trial and error*, *an atomic reactor cannot*.” [1].

The biggest gains in cognitive improvement will most likely first materialize through prosthetics aimed at individuals in whom cognitive function is diminished by injury or disease. Indeed, a flourishing number of recent papers pairing invasive recordings of brain activity (increasingly taking advantage of single-cell resolution) with electrical stimulation via implanted electrodes, machine learning, or both, have demonstrated improvements that might soon pave the way for cognitive neuroprosthetics [5,6]. However, to be considered an enhancer, neurotechnology must produce beneficial effects in the absence of a deficit. But what is the level of enhancement that justifies the risks associated with brain surgery? Noninvasive neurotechnology is objectively the most desired path.

Currently most promising technologies for cognitive enhancement include brain–computer interfaces and brain stimulation. Noninvasive brain stimulation includes technologies with the ability to influence neural activity without the need for surgery. These include techniques that shape brain activity through the generation of local electric fields (i.e., transcranial magnetic or electric stimulation), mechanical interactions (i.e., transcranial focused ultrasound), and sensorial stimulation (e.g., via rhythmic entrainment or closed-loop auditory stimulation). Protocols using noninvasive brain stimulation for cognitive enhancement have been advancing through trial and error for decades, alongside an increased recognition of the large parameter space that needs to be accounted for to optimize stimulation for cognitive functions [3]. Brain stimulation approaches have progressively being refined through modeling, implementation of protocols inspired by the brain’s intrinsic patterns of activity, better engagement with the target area afforded by improving focality, reaching deeper brain areas, and the combination with imaging for personalized protocols. Brain–computer or brain–machine interfaces are systems that translate signals from the brain for use in an external device. Currently, most brain–machine interfaces use sensorimotor signals as inputs, but there are interesting developments in cognitive brain–machine interfaces. These have so far been mostly circumscribed to neurofeedback approaches, with examples of cognitive enhancements targeting attention, memory, and executive functions, and interesting developments in brain-to-brain interface technology, which could be used for social networks and collective decision-making [7]. However, evidence for clear cognitive enhancement via noninvasive brain stimulation or machine-based interfaces is modest and it is still unclear whether long-term benefits that provide competitive advantages in ecological settings can be achieved.

Progress is expected to come through technologies that can make use of closed-loop approaches that “read” and can influence brain activity associated with the cognitive function to be enhanced. While considerations such as high-fidelity neural sensors that can be used for prolonged periods of time, computing power, advanced signal processing, and machine learning techniques are all critical, the biggest challenge lies in the intrinsic complexity of the neural mechanisms underlying cognition. The metaphor of the brain as a machine can be useful and has enabled significant progress. However, the brain is ultimately not a machine, and the biological reductionism that has served as the substrate of many interventions using noninvasive brain stimulation or brain–machine interfaces likely also underlies the lack of robustness and replicability of many of the current findings [8]. If cognitive functions are associated with distributed patterns across brain networks, for which there is no one-to-one mapping, where causal factors interact in nonlinear ways to produce larger-scale collective outcomes [9], and for which the response to a target input is non-stationary, then new paradigms better suited to analyzing and interpreting the brain signals that can be read and the effects on behavior that are likely to be produced will need to be explored and embraced.

Some progress is being made in the fields of neuroscience and neuroimaging through a burgeoning interdisciplinary engagement with the challenges and questions posed by the science of complex systems. This generates both new challenges and new potential. If neurotechnology is to move beyond the limitations of a trial and error approach, it is time to engage with the brain’s irreducible complexity.
